# The Human Gut Microbial Metabolome Modulates Fungal Growth via the TOR Signaling Pathway

**DOI:** 10.1128/mSphere.00555-17

**Published:** 2017-12-13

**Authors:** Carlos García, Faiza Tebbji, Michelle Daigneault, Ning-Ning Liu, Julia R. Köhler, Emma Allen-Vercoe, Adnane Sellam

**Affiliations:** a Infectious Diseases Research Centre-CRI, CHU de Québec Research Center (CHUQ), Université Laval, Quebec City, Quebec, Canada; b Department of Molecular and Cellular Biology, University of Guelph, Guelph, Ontario, Canada; c Division of Infectious Diseases, Boston Children’s Hospital/Harvard Medical School, Boston, Massachusetts, USA; d Department of Microbiology, Infectious Disease and Immunology, Faculty of Medicine, Université Laval, Quebec City, Quebec, Canada; Carnegie Mellon University

**Keywords:** antifungal activity, *Candida albicans*, gut microbial metabolome, TOR pathway

## Abstract

*Candida albicans* is well known as a major human fungal pathogen, but it is also a permanent resident of healthy gastrointestinal tracts. Recent studies have shown that the human gut microbial metabolome represents an interesting source of bioactive molecules with a significant degree of chemical diversity. Some of these bioactive molecules may have useful antivirulence activities. For instance, intestinal bacterial species belonging to the *Lachnospiraceae* family were found to secrete molecules that attenuate *Salmonella* pathogenicity and repress the expression of virulence genes. Here, we have investigated whether the microbial gut metabolome (GM) contains molecules that might promote the commensal lifestyle and/or inhibit the expression of virulence of *C. albicans* in the intestine. We found that metabolites from human feces inhibited the growth of *C. albicans* and other opportunistic yeasts. A genetic screen in *C. albicans* suggested that TOR is the molecular target of the antifungal molecule(s) of the GM. In addition, we found that the GM metabolites inhibit both *C. albicans* hyphal growth and the invasion of human enterocytes. The antigrowth and antivirulence activities were partially recapitulated by secretions from *Roseburia* spp. and *Bacteroides ovatus* strains, respectively. This study demonstrates that the antimicrobial activity of the GM can be extended to a eukaryotic pathogen, *C. albicans*, illuminating the antagonistic interkingdom interactions between a fungus and intestinal commensal bacteria.

**IMPORTANCE** *Candida albicans* is a natural component of the human microbiota but also an opportunistic pathogen that causes life-threatening infections. The human gastrointestinal tract is the main reservoir of *C. albicans*, from where systemic infections originate as a consequence of the disruption of the intestinal mucosal barrier. Recent studies provided convincing evidence that overgrowth of *C. albicans* and other related species in the gut is predominantly associated with chronic intestinal inflammatory bowel diseases. Here, we showed, for the first time, the antagonistic interkingdom interactions between *C. albicans* and common intestinal commensal bacteria. From a therapeutic perspective, administering a defined bacterial community, such as the one described here with anti-*Candida* activity, could provide potential therapeutic protection against gastrointestinal inflammatory diseases.

## Abstract (precis)

*Candida albicans* is a natural component of the human microbiota but also an opportunistic pathogen that causes life-threatening infections. The human gastrointestinal tract is the main reservoir of *C. albicans*, from where systemic infections originate as a consequence of the disruption of the intestinal mucosal barrier. Recent studies provided convincing evidence that overgrowth of *C. albicans* and other related species in the gut is predominantly associated with chronic intestinal inflammatory bowel diseases. Here, we showed, for the first time, the antagonistic interkingdom interactions between *C. albicans* and common intestinal commensal bacteria. From a therapeutic perspective, administering a defined bacterial community, such as the one described here with anti-*Candida* activity, could provide potential therapeutic protection against gastrointestinal inflammatory diseases.

## INTRODUCTION

*Candida albicans* is a natural component of the human microbiota but also an opportunistic pathogen that causes life-threatening infections in immunosuppressed patients. The human gastrointestinal (GI) tract is the main reservoir of *C. albicans*, from where systemic infections originate as a consequence of the disruption of the intestinal mucosal barrier (1, 2). While the mycobiota represent only 0.01 to 0.1% of the human microbiota (3, 4), recent evidence supports the idea that, in certain pathologies, this small fraction can alter the microbiota equilibrium, leading to dysbiosis disease. Of note, overgrowth of *C. albicans* and other *Candida* opportunistic species in the gut is predominantly associated with chronic intestinal inflammatory bowel diseases (IBDs), including Crohn’s disease (CD) and ulcerative colitis (UC) (5). CD in adults is associated with gut microbial dysbiosis characterized by a higher abundance of either *C. albicans* (6, 7), *Candida tropicalis* (8), or *Candida glabrata* (9), while pediatric patients with IBD were found to have increased amounts of *Candida parapsilosis* and *Candida guilliermondii* (10).

The gut microbiota is a complex community of microorganisms that is essential for the development of the host immune system and consequently plays a pivotal role in health and susceptibility to disease. For example, many bacterial communities directly control the activity of the immune system through the production of short-chain fatty acids (SCFAs; e.g., propionate, butyrate, and acetate) which reduce inflammation or modulate the recruitment and maturation of different immune cells (11). Furthermore, the commensal bacterial microbiota contributes to intestinal homeostasis by directly impairing the virulence traits of many bacterial pathobionts. Recent work has demonstrated that metabolites produced by certain species of *Lachnospiraceae*, a bacterial family that is prevalent in the human gut, are able to reduce the growth and silence the expression of invasion genes of *Salmonella enterica* that are necessary to promote infection by this pathogen (12). A similar antivirulence activity against *Salmonella* was reported for butyrate produced by many enteric bacteria, including species belonging to genera such as *Roseburia* and *Faecalibacterium* (13). In addition, commensal bacteria not only suppress the expression of virulence factors of many pathogens but can also preferentially consume nutrients that are required for the growth of competing surrounding pathobionts (14). While convincing evidence supports the idea that the growth and virulence of bacterial pathobionts are controlled by commensal bacteria, little is known regarding how intestinal fungal opportunist species such as *C. albicans* are influenced by other microbial entities. For example, evidence supporting the overgrowth of *Candida* species as a result of the depletion of beneficial commensal microbes with antifungal properties remains unclear, particularly in the context of IBDs.

Many synergistic or antagonistic *C. albicans*-bacterium interactions in different host niches and their clinical impact on human health have been well documented (15). However, so far, such interactions have not been reported for the GI tract in the context of either health or disease. Previous work has shown that sodium butyrate inhibited both growth and filamentation of *C. albicans in vitro* at a concentration similar to that encountered in the gut, providing indirect evidence of *C. albicans* growth control by butyrate-producing bacteria (16). More recently, it was shown that the microbial metabolome of the mammalian gut contains a tremendous number of diverse molecules with critical biological functions (17, 18). We therefore hypothesized that the human gut metabolome (GM) contains molecules that might promote the commensal versus the pathogenic lifestyle of *C. albicans* and other opportunistic fungi. In the current study, we show that metabolites from extracts of human feces exert an antifungal activity against *C. albicans* and other intestine-resident yeasts. This anti-*Candida* activity was recapitulated using fecal extracts derived from other human donors, suggesting a pervasive antifungal feature of these bioactive molecules. In an attempt to gain insight into the mechanism of action associated with the antifungal activity of GM, a genetic screen was undertaken. The obtained data indicated that the GM inhibits the *C. albicans* TOR pathway, a central signaling circuit that controls cellular growth in response to environmental nutrient status in eukaryotes. We also showed that the GM has antivirulence activity through inhibition of both hyphal growth and the invasion of human colon epithelial cells by *C. albicans*. These anti-*Candida* growth and antivirulence activities were partially replicated by exposure of fungal cells to *Roseburia* spp. and *Bacteroides ovatus*, respectively. Further studies assessing the usage of these bacterial species as potential probiotics to restore the microbiota in intestinal pathologies associated with increased abundance of fungal communities represent an exciting direction for future research.

## RESULTS AND DISCUSSION

### The human gut metabolome exerts antifungal activity against *C. albicans*.

**FIG 1:**
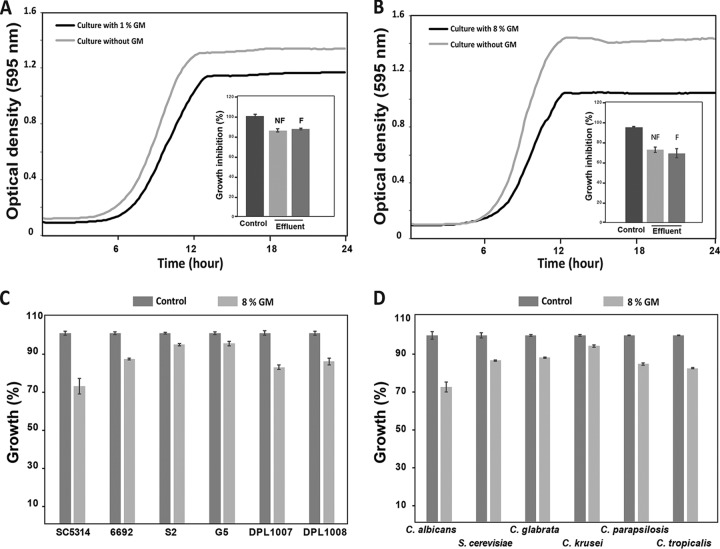
Antifungal activity of the gut secreted metabolome. (A and B) The *C. albicans* SC5314 strain was grown in SC medium supplemented with 1% (A) and 8% (B) bioreactor effluent (DEC60 gut metabolome [GM]). Cells were grown at 30°C, and an OD_595_ reading was taken every 10 min. OD measurements for each GM concentration and control (cells treated with uninoculated bioreactor growth medium) are provided as the mean from triplicate assays. To determine whether the bioactive antifungal molecules were secreted by the components of the microbiota, spent culture medium was separated from bacterial cells by centrifugation followed by supernatant filtration. The histogram in each panel indicates OD_595_ after 24 h of exposure. (C) Antifungal activity of the GM on clinical *C. albicans* azole- (6692, S2, and G5) and echinocandin-resistant (DPL-1007 and DPL-1008) strains. (D) The GM inhibits the growth of representative strains of gut-resident yeasts, including *C. glabrata*, *C. parapsilosis*, *C. krusei*, and *S. cerevisiae*. For panels C and D, cells were grown in SC medium with 8% GM or control growth medium and OD_595_ readings were taken after 24 h of incubation at 30°C under agitation. Results represent mean growth inhibition (percent) after 24 h of treatment of at least three replicates. NF, nonfiltered; F, filtered.

There is growing evidence that gut-resident microbiota control and restrain the growth of gastrointestinal pathogenic bacteria directly by inhibiting their growth or their virulence factors and thus represent a reinforcement of the human immune system (12, 19–21). The antimicrobial activity of the gut microbiota is mediated by their rich secreted metabolome, which has been shown to exhibit a significant chemical diversity (12, 17, 18). We aimed to investigate whether the GM exerts antifungal activity against the major human fungal pathogen *C. albicans*, also a resident of the GI tract. As a source of gut metabolites, we used a continuous-culture bioreactor system to grow a defined microbial community of 60 strains (DEC60) derived from the feces of a healthy human donor (donor 1) (12). Antifungal activity of the GM on *C. albicans* clinical reference strain SC5314 was evaluated by monitoring the optical density at 595 nm (OD_595_) of cultures exposed for 24 h to increased concentrations of the GM. A significant inhibition of *C. albicans* growth was noticed at 1% GM with 15% growth reduction (Fig. 1A and B). An equivalent inhibitory rate was obtained with filtrate from the bioreactor effluent filtered to remove cellular material, suggesting that the GM antifungal activity is mediated by secreted molecules (Fig. 1A and B). This result demonstrated that, in addition to their activity against pathogenic bacteria, metabolites secreted by certain human gut-derived microbes possess antifungal activity against the opportunistic yeast *C. albicans*.

We also tested the GM antifungal activity on resistant *C. albicans* clinical isolates. A total of three fluconazole-resistant strains with different resistance mechanisms (see details in Table S1 in the supplemental material) were selected in addition to two echinocandin-resistant isolates. As with the *C. albicans* SC5314 sensitive strain, the GM inhibited the growth of all tested resistant clinical isolates (Fig. 1C). This demonstrates that the GM contains bioactive molecules that can potentially be used to tackle therapeutic limitations related to acquired clinical resistance to commonly used antifungals. This also suggests that the mechanisms that confer resistance to azoles and echinocandins are distinct from those that may cause resistance to antifungal gut metabolites.

10.1128/mSphere.00555-17.2 TABLE S1  Fungal strains used in this study. Download TABLE S1, DOCX file, 0.1 MB. Copyright © 2017 García et al. 2017 García et al. This content is distributed under the terms of the Creative Commons Attribution 4.0 International license.

### The human gut metabolome exerts a broad antifungal activity against intestine-resident yeasts.

In addition to *C. albicans*, the human gut mycobiome includes other non-*albicans* opportunist *Candida* species, such as *C. glabrata*, *C. tropicalis*, *C. krusei*, and *C. parapsilosis* (22). We tested whether the antifungal activity of the intestinal microbial metabolome can be expanded to these yeasts in addition to the foodborne gut-resident yeast *Saccharomyces cerevisiae*. The obtained data demonstrate that the GM inhibited the growth of all tested fungal species, with *C. albicans* exhibiting the highest sensitivity, followed by *C. tropicalis*, *C. parapsilosis*, *S. cerevisiae*, *C. glabrata*, and *C. krusei* (Fig. 1D). Thus, the GM antifungal activity is generalized to prevalent yeast residents of the gut. An obvious focus for future experimentation will be the investigation of whether the GM antifungal activity has been lost in CD and other inflammatory bowel pathologies associated with the disturbance of the fungal microbiota (23).

### The inhibitory effect of the human gut metabolome is pervasive.

**FIG 2:**
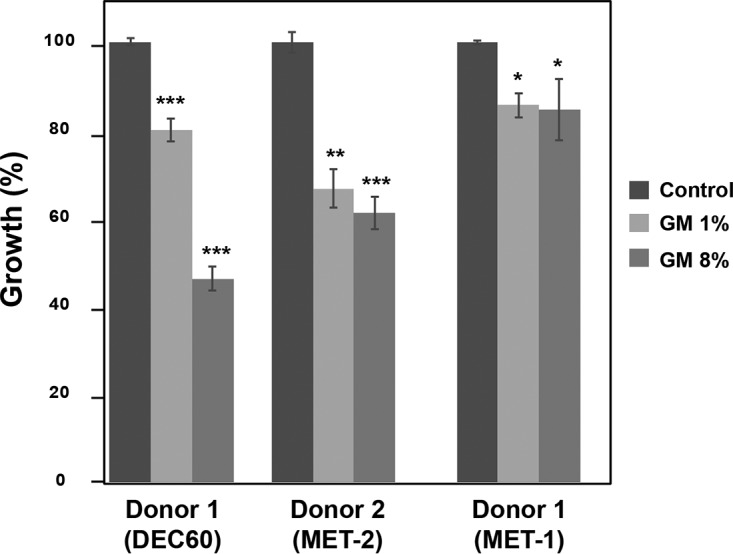
The inhibitory effect of the human gut metabolome is widespread. For each human donor, results represent the average from three independent *C. albicans* cultures. *C. albicans* SC5314 cells were grown in SC medium with 1 and 8% GM or control medium, and an OD_595_ reading was taken after 24 h of incubation at 30°C under agitation. MET-1 is a defined therapeutic microbial ecosystem of 33 bacterial strains from donor 1 (DEC60). Bars show the means ± standard errors of the means. *, *P* < 0.02; **, *P* < 0.01; ***, *P* < 0.0003.

Since the human microbiome is very dynamic and is influenced by different conditions, including the diet and host physiology, we wanted to determine whether or not GM antifungal activity was restricted to the donor used for the above-described experiments. The activity of microbial metabolites derived from defined communities from feces of an additional donor (donor 2) in addition to the therapeutic microbial ecosystem MET-1 (20) from donor 1 was tested on *C. albicans* (Fig. 2). Exposure of the *C. albicans* SC5314 strain to 1% and 8% GM from donor 2 and MET-1 resulted in a significant growth reduction, demonstrating that the biological activity of the gut metabolome is a widespread feature. MET-1 is a therapeutic microbial ecosystem of 33 bacterial strains which has been used to cure recurrent *Clostridium difficile* infection (20). MET-1 partially recapitulated the activity of the GM from DEC60 (donor 1), suggesting that the antifungal activity of the GM may be mediated by several other strains.

### The gut metabolome inhibits *C. albicans* growth through the TOR pathway.

**FIG 3:**
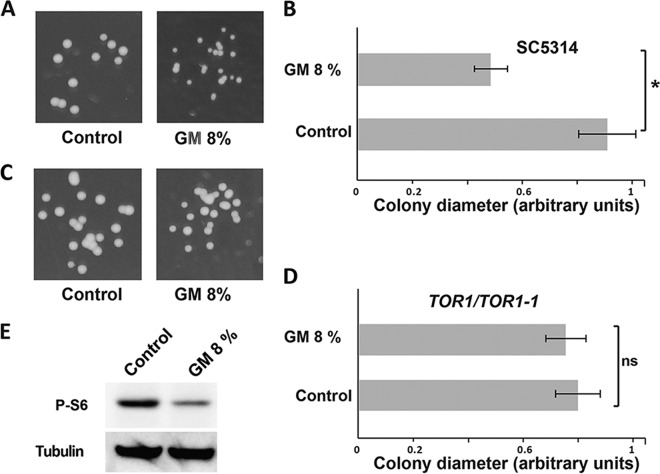
The gut metabolome inhibits *C. albicans* growth through the TOR pathway. (A) In solid SC-agar medium, cells treated with 8% GM resulted in colonies with reduced size. (B) Diameters (measured in arbitrary units) of at least 50 colonies grown in SC-agar with or without GM (8%) were measured. (C) Representative image of the sirolimus-resistant *TOR1-1* strain (JRB12) colony size. (D) Mean diameter of *TOR1-1* colonies. In panels B and D, bars represent the means ± standard errors of the means. *, *P* < 0.00001; ns, not significant (*P* > 0.12). (E) The GM reduces the phosphorylation level of the TOR effector, ribosomal protein S6. *C. albicans* cells were grown in SC medium and treated for 1 h with 8% GM. The same volume of culture medium was added to the control culture. Cell lysates were probed for P-S6 and tubulin (loading control) using anti-phosphorylated-Akt substrate and antitubulin antibodies, respectively. At least three biological replicates were obtained for each experiment shown.

To gain insight into the mechanism of action of the GM associated with its antifungal activity, we screened the *C. albicans* GRACE (Gene Replacement and Conditional Expression) conditional mutant collection (24). In solid medium, *C. albicans* growth inhibition by 8% GM is reflected by a reduction in colony size by almost 50% compared to the control assay (Fig. 3A and B). The colony size was used then as a phenotypic readout to identify genes in *C. albicans* that are required to tolerate or resist the antifungal property of the GM. Among the 2,360 unique mutants screened, a growth defect was reported and confirmed exclusively for the *kog1* mutant. For this strain, growth was inhibited to 60% of that of the wild-type parental strain (not shown). Kog1 is a conserved subunit of the TOR (target of rapamycin) pathway, a central signaling circuit that controls cellular growth in response to environmental nutrient status and stress in eukaryotes (25).

Our finding suggests that the *C. albicans* TOR pathway modulates the cellular response required to tolerate the GM inhibitory effect. To examine whether the GM acts directly on the TOR pathway, the effect of GM was assessed in cells expressing a sirolimus resistance allele of the Tor1 kinase, *TOR1-1* (26). This allele encodes a mutation (S1984R) that confers complete resistance to the growth-inhibitory effect of sirolimus. The colony size of the *TOR1-1*/*TOR1* strain treated with 8% GM was not discernible from the control experiment, suggesting that this strain is impervious to the antifungal activity of the GM (Fig. 3C and D). These data support the idea that the TOR pathway might be a direct target of the antifungal molecule(s) secreted by the gut microbiota.

To further confirm this finding, the effect of GM on the phosphorylation state of the ribosomal protein S6 (P-S6), a conserved effector of the TOR pathway in *C. albicans* (27) and other eukaryotes, was evaluated. We found that the P-S6 phosphorylation level was significantly reduced in GM-treated cells compared to the control assay (Fig. 3E). Under nutrient sufficiency, TOR activates energy-consuming biosynthetic pathways such as ribosome biogenesis, transcription, and translation (28, 29). Oppositely, under starvation or in the presence of sirolimus, TOR activity is depleted, which prevents all processes that are energy consuming and leads the cells to enter into a quiescent state also referred to as G0 (29, 30). Taken together, our data support a model whereby gut microbial cohabitants repress *C. albicans* TOR activity to mimic a starved environment which could lock the cells into a quiescent/G0 state and consequently lower their nutritional competitiveness in the GI tract.

### The transcriptional profile of *C. albicans* cells challenged with the GM resembles nutrient starvation and TOR pathway inhibition.

As shown above, the microbiota-secreted metabolites restrained the growth of *C. albicans* through the alteration of TOR pathway activity. Therefore, we anticipate that *C. albicans* cells exposed to the GM might display a transcriptional signature reminiscent of TOR inhibition, such as that exhibited in response to sirolimus (31). To test this hypothesis, we used RNA sequencing (RNA-seq) to assess the transcriptome of cells treated with 8% GM for 15 min. Gene ontology analysis showed that transcripts related to biosynthesis of amino acids, including arginine, methionine, serine, aspartate, and glutamine, were induced (Table 1). Genes of the nitrogen catabolite repression (NCR) pathway, such as those for ammonium and amino acid permeases, together with the master regulator of nitrogen utilization, Gat1, and the ammonium transport regulator, Npr1 kinase, were also upregulated (Table S2). Of note, activation of both amino acid metabolism and the NCR transcripts has been previously shown to be the bona fide transcriptional signature that *C. albicans* cells and other fungi experience when growing under conditions of nitrogen starvation (32–36) and suggests that exposure to the GM mimics a nitrogen-depleted environment.

10.1128/mSphere.00555-17.3 TABLE S2  List of GM-upregulated transcripts. Download TABLE S2, DOCX file, 0.1 MB. Copyright © 2017 García et al. 2017 García et al. This content is distributed under the terms of the Creative Commons Attribution 4.0 International license.

**TABLE 1:** Gene functions and biological processes associated with *C. albicans* response to GM^b^

GO category	Gene name	*P* value^a^
Upregulated transcripts		
Alpha-amino acid biosynthesis	*ALT1*, *ARG3*, *ARG5* and -*6*, *ARG8*, *CYS1*, *CPA1*, *CPA2*, *DFR1*, *GDH3*, *HAL21*, *HBR2*, *HOM3*, *IDP1*, *LYS12*, *LYS22*, *LYS4*, *MET15*, *MET16*, *MET2*	4.79e−8
Aspartate family amino acid biosynthesis	*CYS1*, *HAL21*, *HOM3*, *LYS12*, *LYS22*, *LYS4*, *MET15*, *MET16*, *MET2*	5.78e−5
Arginine metabolism	*ARG3*, *ARG5* and -*6*, *ARG8*, *CAR2*, *CPA1*, *CPA2*	6e−4
Sulfur amino acid metabolism	*ARO10*, *C1_02970W_A*, *CYS1*, *HAL21*, *HOM3*, *LAP3*, *MET15*, *MET16*, *MET2*	8.4e−4
Glutamine family amino acid biosynthesis	*ARG3*, *ARG5* and -*6*, *ARG8*, *CPA1*, *CPA2*, *GDH3*, *IDP1*	1.33e−3
Carbohydrate metabolism	*ADH2*, *ARA1*, *ATC1*, *GLG21*, *C3_03410C_A*, *C4_02620C_A*, *CIT1*, *DAK2*, *GLK1*, *GLK4*, *GRE3*, *HSP104*, *HSP21*, *HXK2*, *MDH1-1*, *MLS1*, *PGM2*, *PHR1*, *PYC2*, *TPS2*, *XKS1*	1.71e−3
Methionine metabolism	*ARO10*, *CYS1*, *HAL21*, *HOM3*, *MET15*, *MET16*, *MET2*	9.55e−3
Serine metabolism	*CYS1*, *DFR1*, *HBR2*, *HOM3*, *MET15*, *MET16*	7.03e−3
Trehalose metabolism	*ATC1*, *HSP104*, *HSP21*, *PGM2*, *TPS2*	5.46e−3
Alpha-amino acid catabolism	*ADH2*, *ALT1*, *ARO10*, *C1_08490W_A*, *CAR2*, *CPA1*, *LAP3*	4.12e−3
Downregulated transcripts		
Ribosome biogenesis	*BMS1*, *RRP40*, *BUD20*, *NOP9*, *RSA3*, *URB1*, *RRP17*, *LRP1*, *ESF1*, *REX4*, *RRB1*, *ECM16*, *RRP5*, *NOP12*, *EBP2*, *PUF6*, *NOP53*, *RRP46*, *SLX9*, *RRP14*, *NOP16*, *RRP43*, *KRI1*, *POP7*, *RSA4*, *RIX1*, *MAK11*, *FAF1*, *NUG1*, *SNU66*, *C5_04910W_A*, *SQT1*, *DHR2*, *TSR4*, *MTR4*, *CHR1*, *TMA23*, *IFU4*, *RCM1*, *UTP23*, *NAF1*, *NOP2*, *KRE33*, *GRC3*, *RRP36*, *TIF6*, *CSI2*, *DBP3*, *DBP8*, *DIP2*, *ECM1*, *ENP2*, *ERB1*, *FYV5*, *GAR1*, *HAS1*, *HIT1*, *IMP4*, *JIP5*, *MRT4*, *NHP2*, *NIP7*, *NMD3*, *NOC2*, *NOC4*, *NOG2*, *NOP1*, *NOP4*, *NOP5*, *NOP6*, *NOP8*, *NSA1*, *PES1*, *POP4*, *PUS7*, *RCL1*, *REI1*, *RPF1*, *RPF2*, *RPL7*, *RPP1*, *RRP6*, *RRP8*, *RRS1*, *SAS10*, *SDA1*, *SPB1*, *SPB4*, *TSR2*, *UTP13*, *UTP20*, *UTP21*, *UTP4*, *YTM1*, *YVH1*	1.89e−70
Ribosome localization	*BUD20*, *NOP9*, *NOP53*, *SLX9*, *RIX1*, *NUG1*, *TIF6*, *ECM1*, *NMD3*, *NOG2*, *RPF1*, *RRS1*, *SDA1*	2.80e−7
snRNA metabolic process	*MTR4*, *PUS7*, *RRP6*, *NAF1*, *GAR1*, *LRP1*, *NHP2*	5.84e−3
tRNA processing	*RPP1*, *DUS3*, *TGS1*, *DUS1*, *SMM1*, *PUS7*, *POP7*, *SEN2*, *TRM5*, *TRM3*, *NOP1*, *DEG1*, *POP4*	9.45e−3
snoRNA processing	*RPP1*, *RRP6*, *LRP1*, *POP7*, *NOP1*, *POP4*, *MTR4*	4.15e−2
GMP metabolic process	*GUA1*, *HPT1*, *GUK1*	7.62e−2

a The *P* value was calculated using hypergeometric distribution, as described on the GO Term Finder website.

b Gene ontology (GO) analysis was performed using GO Term Finder.

Carbohydrate genes comprising those for hexose transporters, carbon utilization, and trehalose metabolism were also activated by the GM (Table 1). The glycolytic genes *GLK1*, *GLK4*, and *HXK2*, encoding hexokinases that catalyze the phosphorylation of glucose or fructose, the first irreversible step in the intracellular metabolism of hexoses, were upregulated. Activation of sugar transporters and carbon utilization genes reflects the idea that the GM exposure might simulate a carbon-limiting environment.

Strikingly, a large proportion of GM-repressed transcripts were associated with functional categories related to protein translation, including ribosome biogenesis, structural components of the small and large subunits of the ribosome, and ribosomal noncoding gene transcription and processing (rRNA, tRNA, snRNA, and snoRNA) (Table 1; see also Table S3). Repression of translation-related genes is a universal signature observed under conditions where the TOR pathway is compromised (31) or in cells growing in nitrogen- or carbon-starved environments (32, 37). TOR inhibition is also characterized by the transcriptional activation of NCR and carbon utilization genes, suggesting that GM treatments in *C. albicans* recapitulate most of the transcriptional readouts caused by TOR inhibition. In the budding yeast, the TOR signaling cascade controls gene expression in response to starvation for either nitrogen or carbon. The parallels to our data set suggest that the bulk of the transcriptional response observed in *C. albicans* cells challenged with the GM is most likely the consequence of TOR pathway inhibition. RNA-seq data provided further supportive evidence for the role of secreted microbiota metabolites in inhibiting the TOR pathway to modulate *C. albicans* proliferation in the GI tract.

10.1128/mSphere.00555-17.4 TABLE S3  List of GM-downregulated transcripts. Download TABLE S3, DOCX file, 0.03 MB. Copyright © 2017 García et al. 2017 García et al. This content is distributed under the terms of the Creative Commons Attribution 4.0 International license.

### The human gut metabolome inhibits yeast-to-hypha transition in *C. albicans* and the inducibility of hypha-specific transcripts.

**FIG 4:**
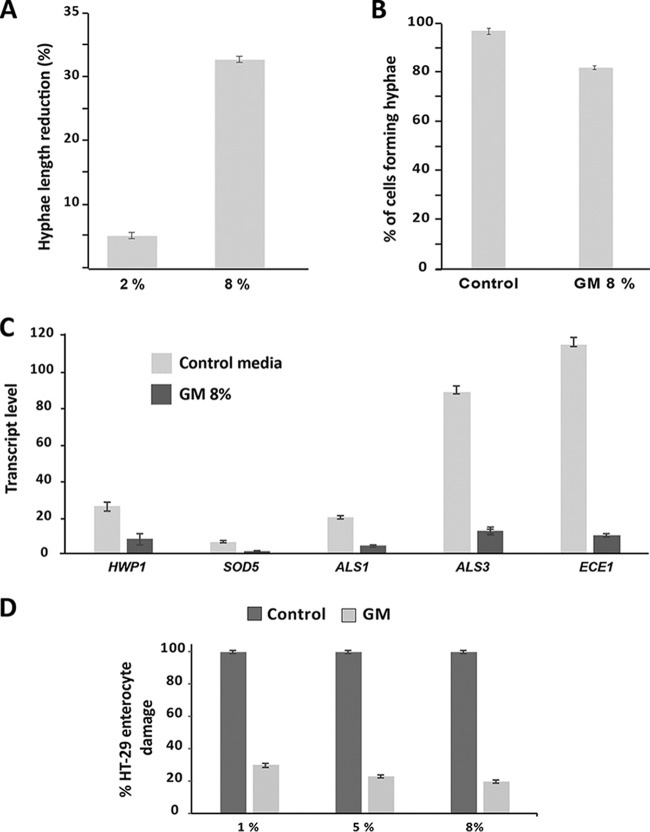
Antivirulence activity of the human gut metabolome. (A and B) The GM inhibits both hypha elongation (A) and hypha formation (B). *C. albicans* SC5314 cells growing at 37°C in the presence of FBS were treated with 2 and 8% GM for 1 h. The number of germ tubes of at least 100 cells treated with GM or not was counted and compared to cells treated with the control culture medium only (B). At the same time, filament lengths were measured and results are presented as the percentage of length reduction compared to the control condition (A). The presented data are representative of three biological replicates. (C) The GM contains molecules that modulate virulence-related gene expression. Transcript levels of bona fide yeast-to-hypha transition genes, including *ALS1*, *ALS3*, *ECE1*, *HWP1*, and *SOD5*, were evaluated in cells exposed to 8% GM for 1 h. Transcript levels were calculated using the comparative *C*_*T*_ method using the *ACT1* gene as a reference. (D) The GM attenuates the damage to human colon epithelial cells (HT-29 cells) caused by *C. albicans*. Damage to HT-29 cells was assessed using an LDH release assay. For each GM concentration, cell damage was calculated as percentage of LDH activity of the GM-treated experiment culture relative to that of the control experiment culture (*C. albicans* invading HT-29 cells in the absence of GM). Results are expressed as the mean from three independent biological replicates.

Previous studies have demonstrated that the gut commensal microbiota produces small molecules with antivirulence activity. For instance, different species of the *Lachnospiraceae* family were found to secrete molecules that attenuate *Salmonella* virulence by repressing expression of the global regulator *hilA*, which controls the expression of *Salmonella* pathogenicity island 1 (SPI1) pathogenicity genes, thereby compromising the pathogen’s ability to invade host cells (12). Since the gut is considered the main reservoir of *C. albicans* and the main source of systemic infection in humans (1), we hypothesized that aspects of a normal gut microbial ecosystem might be required to control *C. albicans* virulence traits. To test this hypothesis, the effect of the GM was assayed by testing the ability of *C. albicans* to form invasive hyphae in response to fetal bovine serum (FBS). Cells exposed to the GM remained responsive to FBS and formed typical hyphae; however, the average length of these hyphae was significantly reduced compared to the control (Fig. 4A). Furthermore, when exposed to 8% GM, only 80% of *C. albicans* cells had differentiated true hyphae compared to nontreated cells (Fig. 4B). At the transcriptional level, hypha-specific genes, including the cytolytic peptide toxin *ECE1*; the superoxide dismutase *SOD5;* and the adhesins *ALS1*, *ALS3*, and *HWP1*, were repressed in cells exposed to the GM (Fig. 4C). Taken together, these results demonstrate that gut microbiota-secreted molecules repress the yeast-to-hypha transcriptional program, which in turn leads to the inhibition of the *C. albicans* invasive form.

### The gut metabolome attenuates damage to intestinal epithelial cells caused by *C. albicans.*

Since the GM reduced the formation of invasive hyphae, we wanted to check whether it conferred a protective activity for host cells against fungal invasion. *C. albicans*-mediated damage of human colon epithelial HT-29 cells was quantified based on the lactate dehydrogenase (LDH) release assay in cells treated or not with three different concentrations of GM (1, 2, and 8%). In accordance with the inhibitory effect on invasive hyphal growth, our data showed clearly that the GM significantly reduced the damage to HT-29 enterocytes (Fig. 4D). Remarkably, the protective effect was seen even at a concentration of 1% GM (70% reduction of enterocyte invasion), which has no impact on hypha formation or elongation (data not shown). Of note, it is well known that microbiota-secreted metabolites such as propionate, acetate, and butyrate positively influence human health by contributing to protection from infection through, e.g., the active recruitment of immune cells (11, 38). For instance, the commensal intestinal bacterium *Bacteroides thetaiotaomicron*, a fermentative anaerobe which produces several SCFAs, induces the expression of the antimicrobial peptide LL-37 in intestinal epithelial cells, in turn protecting from *C. albicans* colonization (39). In this regard, our data suggest that the GM might have a dual action both by directly inhibiting *C. albicans* growth and virulence and by augmenting GI mucosal immunity through SCFA activation to reduce fungal infections.

### Identification of microbial species secreting antifungal inhibitory molecules.

**FIG 5:**
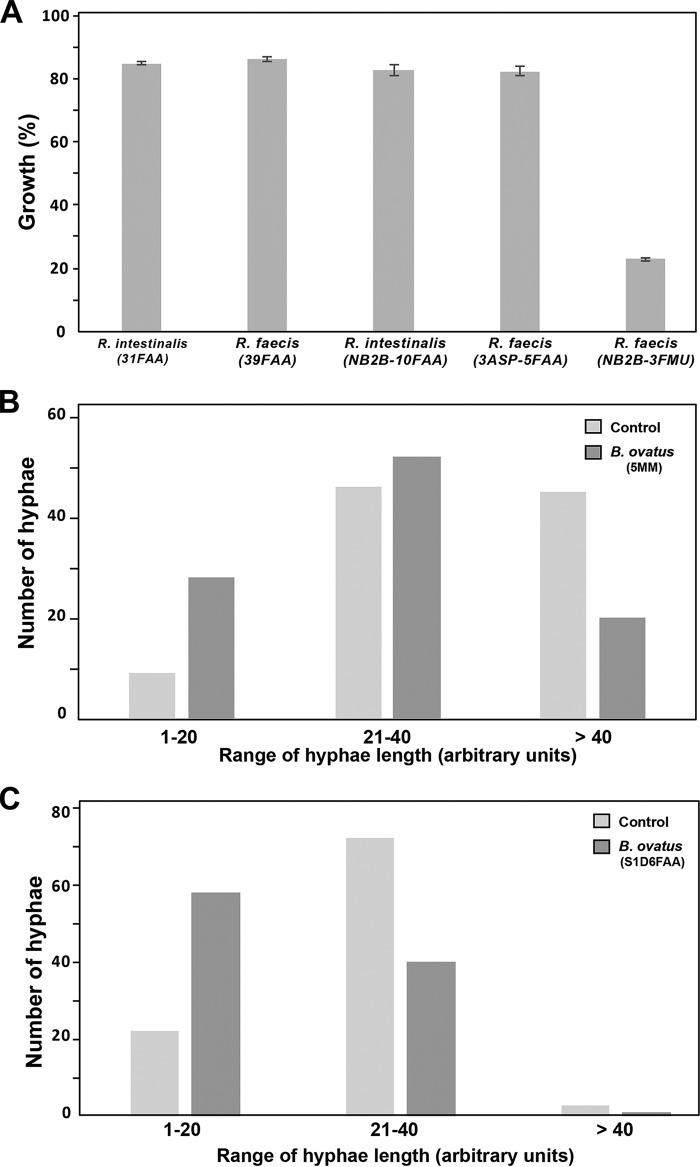
Identification of bacterial species producing the anti-*Candida* molecules. (A) Both *Roseburia intestinalis* and *R. faecis* secreted molecules exert antifungal activity. *C. albicans* cells were grown in SC medium with 8% GM or control medium, and OD_595_ readings were taken after 24 h of incubation at 30°C under agitation. Results represent growth inhibition (percent) after 24 h of treatment of at least three replicates. (B and C) Both *Bacteroides ovatus* strain 5MM (B) and strain S1D6FAA (C) reduced *C. albicans* hypha elongation. A total of 100 cells with three hypha length ranges (1 to 20, 21 to 40, and >40), measured in arbitrary units, were counted. Results are presented as the number of cells with a hypha length in the corresponding range.

To identify the microbial species that produce an antifungal molecule(s), individual isolates from a continuous-culture bioreactor supporting the defined community MET-1 derived from donor 1 were screened for their abilities to inhibit growth of *C. albicans*. Filtered effluents from a total of 33 isolates corresponding to 26 different bacterial species were screened (Table S4). Most of these tested effluents had little or no apparent growth inhibition properties against *C. albicans*. Notable exceptions were effluents from strains belonging to species of the genus *Roseburia*, including *R. faecis* (31FAA) and *R. intestinalis* (39FAA), which exhibited significant antifungal activity against *C. albicans* (15% and 13% growth reduction, respectively [Fig. 5A]). Effluents from further strains of *R. faecis* and *R. intestinalis* sourced from different donors were also tested, and the results confirmed a general anti-*Candida* activity originating from these species (Fig. 5A), suggesting that secreted bioactive molecules of *R. faecis* and *R. intestinalis* contribute to the antifungal activity of the GM.

10.1128/mSphere.00555-17.5 TABLE S4  Bacterial strains used in this study. Download TABLE S4, XLSX file, 0.03 MB. Copyright © 2017 García et al. 2017 García et al. This content is distributed under the terms of the Creative Commons Attribution 4.0 International license.

Interestingly, all species of the *Roseburia* genus described to date are known to produce butyrate using the butyryl coenzyme A (CoA):acetate CoA-transferase fermentation pathway (40, 41). Butyrate is a short-chain fatty acid histone deacetylase (HDAC) inhibitor that plays a critical role in human health (11). However, the anti-*Candida* activity of the GM is unlikely to be related to butyrate, since the effluent of other known butyrate-producing bacteria from donor 1 did not exhibit any apparent activity against *C. albicans* (see complete listing of tested strains and their respective species in Table S5). Pure butyrate concentrations equivalent to the measured levels present in the GMs from donors 1 and 2 (0.05 to 0.25 mM in DEC60, MET-1, and MET-2) were assayed for their ability to inhibit filamentation, colony size, and the growth of *C. albicans* cells in liquid media. As expected, and previously shown (16), butyrate significantly reduced hypha formation of *C. albicans* at >20 mM; however, no apparent effect was noticed at a concentration less than 1 mM (data not shown). Neither growth nor colony size was affected by 0.1 or 0.2 mM butyrate exposure (Fig. S1). Taken together, these data suggest that butyrate alone was not responsible for the GM antifungal activity determined in this study.

10.1128/mSphere.00555-17.1 FIG S1  Effect of sodium butyrate on *C. albicans* growth and colony size. (A) The *C. albicans* SC5314 strain was grown in SC medium supplemented with different concentrations of sodium butyrate. The histogram represents growth inhibition (%) after 24 h of treatment. (B) Effect of different concentrations of sodium butyrate on colony size. Data represent colony size reduction in percentage compared to nontreated *C. albicans* control cells. Download FIG S1, TIF file, 0.8 MB. Copyright © 2017 García et al. 2017 García et al. This content is distributed under the terms of the Creative Commons Attribution 4.0 International license.

10.1128/mSphere.00555-17.6 TABLE S5  Butyrate-producing bacteria from MET-1 defined community. Download TABLE S5, XLSX file, 0.02 MB. Copyright © 2017 García et al. 2017 García et al. This content is distributed under the terms of the Creative Commons Attribution 4.0 International license.

While the growth-inhibitory effect of the gut-secreted metabolome was at least partially mediated by *R. faecis* and *R. intestinalis*, effluents from these bacteria had no effect on *C. albicans* filamentation (data not shown). As in the growth assay, effluents from the 33 bacterial isolates from MET-1 were tested for their antifilamentation activity. Among the tested bacteria, effluent from *Bacteroides ovatus* isolate 5MM exhibited a significant reduction of filament length (Fig. 5B). This finding was confirmed using spent medium from a further *B. ovatus* strain (S1D6FAA) isolated from donor 2 (Fig. 5C). *B. ovatus* is a common component of the resident human gut microbiota thought to play an important role in deriving energy from the indigestible dietary fibers of consumed vegetables (42). Our finding suggests that, in the intestine, *C. albicans* virulence may be suppressed by secreted molecules from *B. ovatus*, thus promoting the commensal lifestyle of this opportunistic fungus.

In conclusion, we showed, for the first time, the antagonistic interkingdom interactions between the opportunistic fungus *C. albicans* and intestinal commensal bacteria *B. ovatus*, *R. intestinalis*, and *R. faecis*. Former studies had demonstrated that administration of a defined microbiota comprising *B. ovatus* in addition to *R. intestinalis* and *R. faecis* helped to cure patients with recurrent *Clostridium difficile* infection and had a protective role against enteric infections by *Salmonella enterica* serovar Typhimurium in a murine model (20, 21). Furthermore, since recent work has demonstrated that the overgrowth of *C. albicans* and other non-*albicans Candida* species is associated with inflammatory bowel disease, and in particular Crohn’s disease (5, 7–10), it may be possible to administer a defined bacterial community with anti-*Candida* activity, including microbes such as *B. ovatus*, *R. intestinalis*, and *R. faecis*, that could provide a potential therapeutic avenue for yeast overgrowth in IBD. This study illustrated a novel biological activity of the secreted intestinal microbial metabolome, and further investigations are required to uncover the chemical nature of the anti-*Candida* molecules secreted by *B. ovatus*, *R. intestinalis*, and *R. faecis*.

## MATERIALS AND METHODS

### Ethics statement.

The Research Ethics Board of the University of Guelph approved the collection of healthy donor stool samples used in this study (REB no. 09AP011). Donor 1 (MET-1 and DEC60) is a healthy female, 41 years old at the time of sample collection (referred to as donor 6 in reference 20). Donor 2 (MET-2 donor) is a healthy male, 44 years old at the time of sample collection (referred to as donor A in reference 18).

### Fungal strains and media.

The fungal reference and clinical strains used in this study are listed and described in Table S1 in the supplemental material. *C. albicans* and the other yeast strains were routinely maintained at 30°C on YPD (1% yeast extract, 2% peptone, 2% dextrose, with 50 mg/ml uridine) or synthetic complete (SC; 0.67% yeast nitrogen base with ammonium sulfate, 2.0% glucose, and 0.079% complete supplement mixture) medium.

### Bacterial strains and media.

A subset of each donor 1 and 2 community was cultured as individual isolates to obtain culture supernatants for specific testing against *C. albicans* strains. The individual strains cultured are listed in Table S4. Each isolate was cultured in prereduced Trypticase soy broth (Oxoid) supplemented with menadione and hemin (each at 5 mg/liter) in a Ruskin Concept 400 anaerobe chamber at 37°C for 48 h under an atmosphere of 5% H_2_, 5% CO_2_, and 90% N_2_. Supernatants were prepared by centrifuging culture for 5 min at 10,000 × *g* and passing the supernatant through an 0.22-μm sterile syringe filter (Sartorius).

### Microbial community growth and metabolome production in bioreactors.

Fecal material or defined microbial communities were cultured in Infors Multifors bioreactor vessels (Infors AG, Switzerland) using parameters and procedures that we have previously described (43). Briefly, 400-ml vessels were operated under continuous-culture conditions set to mimic the distal human gut: 37°C, pH 7, gently agitated, oxygen free, and fed with a constant supply of mucin (4 g/liter) and insoluble starch substrates (12 g/liter) at a flow rate of ~400 ml/day. Vessels were anaerobically maintained by constant bubbling of N_2_ gas. A sample of culture from the bioreactor was withdrawn for this work once the vessel had reached steady state (21 days postinoculation for the fecal community and 10 days postinoculation from the defined communities). For this work, a fecal community was derived from a stool sample of donor 2 by obtaining a fresh fecal sample from the donor in a sealed plastic container, which was placed within the anaerobe chamber within 10 min of defecation. A 10% (wt/vol) fecal slurry was obtained by mixing 5 g of the homogenized fecal sample into 50 ml of prereduced chemostat feed medium with a stomacher (Tekmar Stomacher Lab Blender; Seward, Worthing, West Sussex, United Kingdom). Once homogenized, the fecal slurry was centrifuged (10 min, 175 × *g*) to remove large particulates, such as residual undigested food. The remaining supernatant was used as fecal inoculum for the bioreactor vessel. Defined communities DEC60 and MET-1 (from donor 1) were prepared for inoculation by preculture of individual isolates on fastidious anaerobe agar (FAA; Acumedia) with the addition of 5% defibrinated sheep’s blood (Hemostat Laboratories), except for *Faecalibacterium prausnitzii*, for which blood was omitted. Isolates were cultured for 48 h in a Ruskin Concept 400 anaerobe chamber, as described above, and biomass was scraped directly into prereduced chemostat growth medium according to formulations indicated in Table S4 and used to inoculate the vessel. Supernatants of vessel samples were prepared by centrifuging culture for 30 min at 15,000 × *g* and passing the supernatant through an 0.22-μm sterile syringe filter (Sartorius).

### Growth inhibition assays.

The antifungal activities of the GM and individual bacterial effluents were evaluated as follows: different fungal strains were grown overnight in YPD medium at 30°C in a shaking incubator. Cells were then suspended in fresh SC medium at an optical density at 595 nm (OD_595_) of 0.05. A total volume of 99 µl or 92 µl of *C. albicans* culture was added to each well of a flat-bottomed 96-well plate in addition to 1 µl or 8 µl of GM, in order to get 1 and 8% GM, respectively. For controls, equivalent sterile, uninoculated bioreactor control medium was used in place of GM. The plates were incubated in the Sunrise-Tecan plate reader at 30°C under agitation, and OD_595_ readings were taken every 10 min over 24 h. All experiments were carried out in triplicate, and average values for each set were calculated for analysis.

### Filamentation assay.

An overnight culture of the *C. albicans* SC5314 strain was used to inoculate 10 ml of fresh YPD at an OD_595_ of 0.05. The cells were grown for 4 h at 30°C under agitation to reach the exponential phase. To induce hypha formation, fetal bovine serum (Life Technologies) was added as a supplement at 10% and cells were incubated at 37°C under agitation for 1 h. The percentage of germ-tube-forming hyphae was determined, and the length of the generated filaments was simultaneously assessed. At least 100 fungal cells were counted per sample, and all experiments were performed in triplicate.

### Genetic screen for gut metabolome-sensitive mutants.

We screened a set of 2,360 strains from the GRACE collection (24), which bear a gene deletion at one locus and an integrated tetracycline-regulated allele at the other locus. Mutant strains were grown overnight on SC medium on flat-bottomed 384-well plates and were plated on SC agar solid medium with or without GM (8%) using a 384-well blot replicator. Colony diameter was measured using the colony imager (spImager; S&P Robotics). Mutants exhibiting more than 2-fold growth reduction based on colony diameter were considered to have been affected by treatment. Each of these mutant phenotypes was then confirmed by spreading a dilution of an overnight culture on an SC medium petri dish to resolve well-separated colonies. The average diameters of at least 50 colonies of each mutant strain as well as the parental wild-type strain were measured. The same procedure was used to measure the colony diameter of the sirolimus-resistant strain JRB12.

### Intestinal epithelial cell damage assay.

Damage to the human colon epithelial cell line HT-29 (ATCC; HTB-38) was assessed using a lactate dehydrogenase (LDH) cytotoxicity detection kit (Sigma), which measures the release of LDH in the growth medium. The manufacturer’s protocol was followed. HT-29 cells were grown as monolayers in Dulbecco’s modified Eagle’s medium (DMEM) supplemented with 10% FBS to 95% confluence in a 96-well tissue culture plate and incubated at 37°C with 5% CO_2_. Prepared cells were infected with 2 × 10^6^ *C. albicans* SC5314 blastospores for 24 h at 37°C with 5% CO_2_. Following incubation, 100 µl supernatant was removed from each experimental well and LDH activity in this supernatant was determined by measuring the absorbance at 490 nm (OD_490_). LDH activity was calculated as the mean from at least four independent biological replicates.

### Expression analysis by real-time quantitative PCR (qPCR).

Saturated overnight culture of the *C. albicans* SC5314 strain was diluted to an OD_595_ of 0.1 in 50 ml fresh SC and grown at 30°C to an OD_595_ of 0.8. The culture was then divided into two equal fractions. Fraction 1 was subjected to hypha induction by supplementing the cultures with 10% fetal bovine serum (FBS) and incubating them at 37°C for 60 min. Fraction 2 was incubated at 30°C for 60 min to encourage the maintenance of the yeast form. GM-treated (8%) and control cultures were harvested by centrifugation at 3,000 × *g* for 5 min, and the pellet was rapidly frozen in liquid nitrogen. Total RNAs were extracted using the Qiagen RNeasy kit as follows: samples stored at −80°C were placed on ice, and RNeasy RLT buffer was added to pellets at a 1:1 (vol/vol) ratio of buffer to pellet. The pellet was allowed to thaw in the buffer and processed with a vortex device briefly at high speed. The resuspended pellet was placed back on ice and divided into 1-ml aliquots in 2-ml screw-cap microcentrifuge tubes containing 0.8 ml of 3-mm-diameter acid-washed glass beads. Samples were homogenized 4 times for 1 min each time in a FastPrep-24 bead beater for 60 s at a speed of 6.5 m/s. Samples were placed on ice for 5 min after each homogenization step. Following homogenization, the Qiagen RNeasy protocol was followed as recommended by the supplier. Total RNA samples were eluted in RNase-free H_2_O. RNA quality and integrity were assessed using an Agilent 2100 Bioanalyzer.

cDNA was synthesized from 2 μg of total RNA using the SuperScript III (Life Technologies) reverse transcription (RT) system [1× RT buffer, 10 mM dithiothreitol, 2.5 mM MgCl_2_, 400 nM oligo(dT)_15_, 1 μM random hexamers, 1 mM deoxynucleoside triphosphate (dNTP), 200 units Superscript III reverse transcriptase]. The total volume was adjusted to 20 μl, and the mixture was then incubated 60 min at 52°C. The RT reaction was terminated by heating at 85°C for 5 min. The RNA template was removed from the cDNA-RNA duplex by adding RNase H at 2 U/µl and incubating the mixture at 37°C for 20 min. Aliquots of the resulting first-strand cDNA were used for real-time PCR amplification experiments. Real-time PCR was performed using the Applied Biosystems 7500 real-time PCR system with the SYBR green PCR master mix (Qiagen) according to the manufacturer’s instructions. After a 10-min denaturation at 95°C, the reaction mixtures were cycled 40 times at 95°C for 15 s, 56°C for 15 s, and 72°C for 30 s. To verify that only the specific product was amplified, a melting point analysis was performed after the last cycle by cooling samples to 55°C and then increasing the temperature to 95°C at 0.2°C per second. A single product at a specific melting temperature was found for each target. All samples were tested in triplicate, and the mean was determined for further calculations. To evaluate the transcript level of the studied genes, the results were normalized using threshold cycle (*C*_*T*_) values obtained from actin (Act1, orf19.5007). The relative quantification analysis was performed using the comparative *C*_*T*_ method (44). Primers used for real-time quantitative PCR (qPCR) are listed in Table 2.

**TABLE 2:** Primer sets used in real-time quantitative PCR

Gene	5′ primer	3′ primer
*ACT1*	GAAGCCCAATCCAAAAGA	CTTCTGGAGCAACTCTCAATTC
*ALS1*	CCTATCTGACTAAGACTGCACC	ACAGTTGGATTTGGCAGTGGA
*ALS3*	CGGTTGCGACTGCAAAGAC	GACCAACCCAAAACAGCATTCC
*HWP1*	CAGTTCCACTCATGCAACCATC	GCAATACCAATAATAGCAGCACCG
*SOD5*	ACGAGGGACACGGCAATGCT	GCGCCATTACCTTGAGGAGCAGTA
*ECE-1*	CCGGCATCTCTTTTAACTGG	GAGATGGCGTTCCAGATGTT

### RNA-seq profiling.

Treatment of *C. albicans* cells with the GM and the RNA extraction procedure were performed as described for the qPCR experiment. A total of 5 µg of total RNA was used for RNA sequencing, and samples were analyzed in duplicate for all conditions. cDNA was generated from total RNA using the Clontech SMARTer PCR cDNA synthesis kit (Clontech). The resulting cDNA was fragmented using Bioruptor (Diagenode, Inc., Denville, NJ, USA), profiled using an Agilent Bioanalyzer, and subjected to Illumina library preparation using SPRIworks HT (Beckman Coulter). The quality and quantity and the size distribution of the Illumina library were determined using an Agilent Bioanalyzer. The library was then submitted for Illumina HiSeq2500 sequencing according to the standard operation. Paired-end 126-nucleotide (nt) reads were generated and checked for data quality using FASTQC (http://www.bioinformatics.babraham.ac.uk/projects/fastqc/). The raw RNA-seq reads for each sample were aligned to the reference *C. albicans* SC5314 genome using STAR v2.4.0 (45) with default parameters. After alignment, estimation of transcript abundance measures as fragments per kilobase of exon per million aligned fragments (FPKM) was performed using Cufflinks in the Tuxedo protocol (46). Differentially expressed transcripts were identified using a fold change cutoff of 2 and a false-discovery rate (FDR) of less than 1%. The complete data set of the RNA-seq experiment is presented in Table S6.

10.1128/mSphere.00555-17.7 TABLE S6  Raw data of RNA-seq experiment. Download TABLE S6, XLSX file, 1.2 MB. Copyright © 2017 García et al. 2017 García et al. This content is distributed under the terms of the Creative Commons Attribution 4.0 International license.

### Ribosomal protein S6 Western blot analyses.

Ribosomal protein S6 phosphorylation assays were performed as described previously (27). Briefly, *C. albicans* SC5314 cells were grown overnight in YPD at 30°C with shaking, washed and resuspended in phosphate-buffered saline (PBS), and diluted into fresh SC medium to a starting OD_595_ of 0.2. Cultures were grown for 2 h before being exposed to 8% GM for an hour. Cells were then harvested by centrifugation and lysed by bead beating in buffer S6 (50 mM Tris Cl, pH 7.5, 150 mM NaCl, 5 mM EDTA, 10% glycerol, 0.2% Nonidet P-40, 1 mM dithiothreitol [DTT], 1 mM phenylmethylsulfonyl fluoride [PMSF], cOmplete Mini EDTA-free protease inhibitor cocktail [Roche Applied Science], 0.1 mM sodium orthovanadate, 20 μM sodium glycerophosphate, 20 μM *para*-nitrophenylphosphate, 20 μM sodium fluoride). Cell lysates were separated by SDS-PAGE and transferred to polyvinylidene difluoride (PVDF) membranes, which were probed for P-S6 using anti-phospho-(S/T)-Akt substrate rabbit polyclonal antibody (Cell Signaling Technology). Antitubulin rat monoclonal antibody (Abcam) was used to monitor the loading controls. Secondary antibodies used were bovine anti-rabbit antibody (Santa Cruz Biotechnology) and goat anti-rat antibody (Abcam). The blots were imaged using a Kodak Image Station 4000 MM. The presented data are representative of at least three biological replicates.

### Statistical analyses.

Fungal growth data were analyzed using one-tailed, unpaired *t* tests.

### Data availability. 

The RNA-seq data are provided in Table S6 in the supplemental material.

## References

[1] Nucci M, Anaissie E 2001 Revisiting the source of candidemia: skin or gut? Clin Infect Dis 33:1959–1967. doi:10.1086/323759. 11702290

[2] Miranda LN, van der Heijden IM, Costa SF, Sousa AP, Sienra RA, Gobara S, Santos CR, Lobo RD, Pessoa VP Jr, Levin AS 2009 Candida colonisation as a source for candidaemia. J Hosp Infect 72:9–16. doi:10.1016/j.jhin.2009.02.009. 19303662

[3] Ott SJ, Kühbacher T, Musfeldt M, Rosenstiel P, Hellmig S, Rehman A, Drews O, Weichert W, Timmis KN, Schreiber S 2008 Fungi and inflammatory bowel diseases: alterations of composition and diversity. Scand J Gastroenterol 43:831–841. doi:10.1080/00365520801935434. 18584522

[4] Underhill DM, Iliev ID 2014 The mycobiota: interactions between commensal fungi and the host immune system. Nat Rev Immunol 14:405–416. doi:10.1038/nri3684. 24854590 PMC4332855

[5] Sam QH, Chang MW, Chai LY 2017 The fungal mycobiome and its interaction with gut bacteria in the host. Int J Mol Sci 18:330. doi:10.3390/ijms18020330. PMC5343866 28165395

[6] Standaert-Vitse A, Sendid B, Joossens M, François N, Vandewalle-El Khoury P, Branche J, Van Kruiningen H, Jouault T, Rutgeerts P, Gower-Rousseau C, Libersa C, Neut C, Broly F, Chamaillard M, Vermeire S, Poulain D, Colombel JF 2009 Candida albicans colonization and ASCA in familial Crohn’s disease. Am J Gastroenterol 104:1745–1753. doi:10.1038/ajg.2009.225. 19471251

[7] Sokol H, Leducq V, Aschard H, Pham HP, Jegou S, Landman C, Cohen D, Liguori G, Bourrier A, Nion-Larmurier I, Cosnes J, Seksik P, Langella P, Skurnik D, Richard ML, Beaugerie L 2017 Fungal microbiota dysbiosis in IBD. Gut 66:1039–1048. doi:10.1136/gutjnl-2015-310746. 26843508 PMC5532459

[8] Hoarau G, Mukherjee PK, Gower-Rousseau C, Hager C, Chandra J, Retuerto MA, Neut C, Vermeire S, Clemente J, Colombel JF, Fujioka H, Poulain D, Sendid B, Ghannoum MA 2016 Bacteriome and mycobiome interactions underscore microbial dysbiosis in familial Crohn’s disease. mBio 7:e01250-16. doi:10.1128/mBio.01250-16. 27651359 PMC5030358

[9] Liguori G, Lamas B, Richard ML, Brandi G, da Costa G, Hoffmann TW, Di Simone MP, Calabrese C, Poggioli G, Langella P, Campieri M, Sokol H 2016 Fungal dysbiosis in mucosa-associated microbiota of Crohn’s disease patients. J Crohns Colitis 10:296–305. doi:10.1093/ecco-jcc/jjv209. 26574491 PMC4957473

[10] Chehoud C, Albenberg LG, Judge C, Hoffmann C, Grunberg S, Bittinger K, Baldassano RN, Lewis JD, Bushman FD, Wu GD 2015 Fungal signature in the gut microbiota of pediatric patients with inflammatory bowel disease. Inflamm Bowel Dis 21:1948–1956. doi:10.1097/MIB.0000000000000454. 26083617 PMC4509842

[11] Sharon G, Garg N, Debelius J, Knight R, Dorrestein PC, Mazmanian SK 2014 Specialized metabolites from the microbiome in health and disease. Cell Metab 20:719–730. doi:10.1016/j.cmet.2014.10.016. 25440054 PMC4337795

[12] Antunes LC, McDonald JA, Schroeter K, Carlucci C, Ferreira RB, Wang M, Yurist-Doutsch S, Hira G, Jacobson K, Davies J, Allen-Vercoe E, Finlay BB 2014 Antivirulence activity of the human gut metabolome. mBio 5:e01183-14. doi:10.1128/mBio.01183-14. 25073640 PMC4128352

[13] Gantois I, Ducatelle R, Pasmans F, Haesebrouck F, Hautefort I, Thompson A, Hinton JC, Van Immerseel F 2006 Butyrate specifically down-regulates *Salmonella* pathogenicity island 1 gene expression. Appl Environ Microbiol 72:946–949. doi:10.1128/AEM.72.1.946-949.2006. 16391141 PMC1352287

[14] Kamada N, Chen GY, Inohara N, Núñez G 2013 Control of pathogens and pathobionts by the gut microbiota. Nat Immunol 14:685–690. doi:10.1038/ni.2608. 23778796 PMC4083503

[15] Morales DK, Hogan DA 2010 Candida albicans interactions with bacteria in the context of human health and disease. PLoS Pathog 6:e1000886. doi:10.1371/journal.ppat.1000886. 20442787 PMC2861711

[16] Nguyen LN, Lopes LC, Cordero RJ, Nosanchuk JD 2011 Sodium butyrate inhibits pathogenic yeast growth and enhances the functions of macrophages. J Antimicrob Chemother 66:2573–2580. doi:10.1093/jac/dkr358. 21911344

[17] Antunes LC, Han J, Ferreira RB, Lolić P, Borchers CH, Finlay BB 2011 Effect of antibiotic treatment on the intestinal metabolome. Antimicrob Agents Chemother 55:1494–1503. doi:10.1128/AAC.01664-10. 21282433 PMC3067180

[18] Yen S, McDonald JA, Schroeter K, Oliphant K, Sokolenko S, Blondeel EJ, Allen-Vercoe E, Aucoin MG 2015 Metabolomic analysis of human fecal microbiota: a comparison of feces-derived communities and defined mixed communities. J Proteome Res 14:1472–1482. doi:10.1021/pr5011247. 25670064

[19] Vogt SL, Peña-Díaz J, Finlay BB 2015 Chemical communication in the gut: effects of microbiota-generated metabolites on gastrointestinal bacterial pathogens. Anaerobe 34:106–115. doi:10.1016/j.anaerobe.2015.05.002. 25958185

[20] Petrof EO, Gloor GB, Vanner SJ, Weese SJ, Carter D, Daigneault MC, Brown EM, Schroeter K, Allen-Vercoe E 2013 Stool substitute transplant therapy for the eradication of Clostridium difficile infection: “RePOOPulating” the gut. Microbiome 1:3. doi:10.1186/2049-2618-1-3. 24467987 PMC3869191

[21] Martz SL, McDonald JA, Sun J, Zhang YG, Gloor GB, Noordhof C, He SM, Gerbaba TK, Blennerhassett M, Hurlbut DJ, Allen-Vercoe E, Claud EC, Petrof EO 2015 Administration of defined microbiota is protective in a murine Salmonella infection model. Sci Rep 5:16094. doi:10.1038/srep16094. 26531327 PMC4632038

[22] Suhr MJ, Hallen-Adams HE 2015 The human gut mycobiome: pitfalls and potentials—a mycologist’s perspective. Mycologia 107:1057–1073. doi:10.3852/15-147. 26354806

[23] Cui L, Morris A, Ghedin E 2013 The human mycobiome in health and disease. Genome Med 5:63. doi:10.1186/gm467. 23899327 PMC3978422

[24] Roemer T, Jiang B, Davison J, Ketela T, Veillette K, Breton A, Tandia F, Linteau A, Sillaots S, Marta C, Martel N, Veronneau S, Lemieux S, Kauffman S, Becker J, Storms R, Boone C, Bussey H 2003 Large-scale essential gene identification in Candida albicans and applications to antifungal drug discovery. Mol Microbiol 50:167–181. doi:10.1046/j.1365-2958.2003.03697.x. 14507372

[25] Loewith R, Hall MN 2011 Target of rapamycin (TOR) in nutrient signaling and growth control. Genetics 189:1177–1201. doi:10.1534/genetics.111.133363. 22174183 PMC3241408

[26] Cruz MC, Goldstein AL, Blankenship J, Del Poeta M, Perfect JR, McCusker JH, Bennani YL, Cardenas ME, Heitman J 2001 Rapamycin and less immunosuppressive analogs are toxic to Candida albicans and Cryptococcus neoformans via FKBP12-dependent inhibition of TOR. Antimicrob Agents Chemother 45:3162–3170. doi:10.1128/AAC.45.11.3162-3170.2001. 11600372 PMC90798

[27] Chowdhury T, Köhler JR 2015 Ribosomal protein s6 phosphorylation is controlled by TOR and modulated by PKA in Candida albicans. Mol Microbiol 98:384–402. doi:10.1111/mmi.13130. 26173379 PMC4631378

[28] Crespo JL, Hall MN 2002 Elucidating TOR signaling and rapamycin action: lessons from Saccharomyces cerevisiae. Microbiol Mol Biol Rev 66:579–591. doi:10.1128/MMBR.66.4.579-591.2002. 12456783 PMC134654

[29] Inoki K, Ouyang H, Li Y, Guan KL 2005 Signaling by target of rapamycin proteins in cell growth control. Microbiol Mol Biol Rev 69:79–100. doi:10.1128/MMBR.69.1.79-100.2005. 15755954 PMC1082789

[30] Gray JV, Petsko GA, Johnston GC, Ringe D, Singer RA, Werner-Washburne M 2004 “Sleeping Beauty”: quiescence in *Saccharomyces cerevisiae*. Microbiol Mol Biol Rev 68:187–206. doi:10.1128/MMBR.68.2.187-206.2004. 15187181 PMC419917

[31] Bastidas RJ, Heitman J, Cardenas ME 2009 The protein kinase Tor1 regulates adhesin gene expression in Candida albicans. PLoS Pathog 5:e1000294. doi:10.1371/journal.ppat.1000294. 19197361 PMC2631134

[32] Ramachandra S, Linde J, Brock M, Guthke R, Hube B, Brunke S 2014 Regulatory networks controlling nitrogen sensing and uptake in Candida albicans. PLoS One 9:e92734. doi:10.1371/journal.pone.0092734. 24651113 PMC3961412

[33] Morschhäuser J 2011 Nitrogen regulation of morphogenesis and protease secretion in Candida albicans. Int J Med Microbiol 301:390–394. doi:10.1016/j.ijmm.2011.04.005. 21555241

[34] Cardenas ME, Cutler NS, Lorenz MC, Di Como CJ, Heitman J 1999 The TOR signaling cascade regulates gene expression in response to nutrients. Genes Dev 13:3271–3279. doi:10.1101/gad.13.24.3271. 10617575 PMC317202

[35] Hardwick JS, Kuruvilla FG, Tong JK, Shamji AF, Schreiber SL 1999 Rapamycin-modulated transcription defines the subset of nutrient-sensitive signaling pathways directly controlled by the Tor proteins. Proc Natl Acad Sci U S A 96:14866–14870. doi:10.1073/pnas.96.26.14866. 10611304 PMC24739

[36] Boer VM, de Winde JH, Pronk JT, Piper MD 2003 The genome-wide transcriptional responses of Saccharomyces cerevisiae grown on glucose in aerobic chemostat cultures limited for carbon, nitrogen, phosphorus, or sulfur. J Biol Chem 278:3265–3274. doi:10.1074/jbc.M209759200. 12414795

[37] Rodaki A, Bohovych IM, Enjalbert B, Young T, Odds FC, Gow NA, Brown AJ 2009 Glucose promotes stress resistance in the fungal pathogen Candida albicans. Mol Biol Cell 20:4845–4855. doi:10.1091/mbc.E09-01-0002. 19759180 PMC2777113

[38] Brestoff JR, Artis D 2013 Commensal bacteria at the interface of host metabolism and the immune system. Nat Immunol 14:676–684. doi:10.1038/ni.2640. 23778795 PMC4013146

[39] Fan D, Coughlin LA, Neubauer MM, Kim J, Kim MS, Zhan X, Simms-Waldrip TR, Xie Y, Hooper LV, Koh AY 2015 Activation of HIF-1alpha and LL-37 by commensal bacteria inhibits Candida albicans colonization. Nat Med 21:808–814. doi:10.1038/nm.3871. 26053625 PMC4496259

[40] Louis P, Flint HJ 2009 Diversity, metabolism and microbial ecology of butyrate-producing bacteria from the human large intestine. FEMS Microbiol Lett 294:1–8. doi:10.1111/j.1574-6968.2009.01514.x. 19222573

[41] Pryde SE, Duncan SH, Hold GL, Stewart CS, Flint HJ 2002 The microbiology of butyrate formation in the human colon. FEMS Microbiol Lett 217:133–139. doi:10.1111/j.1574-6968.2002.tb11467.x. 12480096

[42] Larsbrink J, Rogers TE, Hemsworth GR, McKee LS, Tauzin AS, Spadiut O, Klinter S, Pudlo NA, Urs K, Koropatkin NM, Creagh AL, Haynes CA, Kelly AG, Cederholm SN, Davies GJ, Martens EC, Brumer H 2014 A discrete genetic locus confers xyloglucan metabolism in select human gut Bacteroidetes. Nature 506:498–502. doi:10.1038/nature12907. 24463512 PMC4282169

[43] McDonald JA, Schroeter K, Fuentes S, Heikamp-Dejong I, Khursigara CM, de Vos WM, Allen-Vercoe E 2013 Evaluation of microbial community reproducibility, stability and composition in a human distal gut chemostat model. J Microbiol Methods 95:167–174. doi:10.1016/j.mimet.2013.08.008. 23994646

[44] Guillemette T, Sellam A, Simoneau P 2004 Analysis of a nonribosomal peptide synthetase gene from Alternaria brassicae and flanking genomic sequences. Curr Genet 45:214–224. doi:10.1007/s00294-003-0479-z. 14727058

[45] Dobin A, Davis CA, Schlesinger F, Drenkow J, Zaleski C, Jha S, Batut P, Chaisson M, Gingeras TR 2013 STAR: ultrafast universal RNA-seq aligner. Bioinformatics 29:15–21. doi:10.1093/bioinformatics/bts635. 23104886 PMC3530905

[46] Trapnell C, Roberts A, Goff L, Pertea G, Kim D, Kelley DR, Pimentel H, Salzberg SL, Rinn JL, Pachter L 2012 Differential gene and transcript expression analysis of RNA-seq experiments with TopHat and Cufflinks. Nat Protoc 7:562–578. doi:10.1038/nprot.2012.016. 22383036 PMC3334321

