# Supplementary material for: The Human Gut Microbial Metabolome Modulates Fungal Growth via the TOR Signaling Pathway
Source: mSphere. 2017 Dec 13;2(6):e00555-17. doi: 10.1128/mSphere.00555-17 (PMC5729221; doi:10.1128/mSphere.00555-17)
Supplement: TABLE S1 [file sph006172431st1.docx]

| **Name** | **Description** | **Reference** |
| --- | --- | --- |
| ***Candida albicans*** | | |
| SC5314  (ATCC-MYA-2876) | *C. albicans* wild-type reference strain. | (Fonzi and Irwin 1993) |
| 6692 | Azole-resistant clinical strain (overexpressing the MFS-transporter MDR1 and had a gain-of-function mutation on the transcription factor, Mrr1) isolated from mouth | (Dunkel et al 2008a, Saidane et al 2006) |
| S2 | Fluconazole-resistant clinical strain isolated from patient with AIDS (resistance related to gain-of-function mutation of the transcription factor, Upc2 and Erg11 overexpression) | (Dunkel et al 2008b, Franz et al 1999) |
| G5 | Azole-resistant clinical strain (overexpressing MDR1 and harbouring a gain-of-function mutation on the transcription factor, Mrr1) isolated from oral cavity from patient with AIDS | (Franz et al 1998, Morschhauser et al 2007) |
| DPL-1007 | Clinical isolate resistant to echinocandin harbouring the F641S mutation on the beta-(1,3)-glucan synthase, Fks1p | (Jimenez-Ortigosa et al 2014) |
| DPL-1008 | Clinical isolate resistant to echinocandin harbouring the F645P mutation on the beta-(1,3)-glucan synthase, Fks1p | (Jimenez-Ortigosa et al 2014) |
| JRB12 | SC5314 TOR1-1/TOR1; A laboratory strain that is resistant to the TOR pathway inhibitor, Rapamycin. | (Cruz et al 2001) |
| ***Saccharomyces cerevisiae*** | | |
| BY4741  (ATCC 4040002) | *S. cerevisiae* laboratory reference strain used as genetic background for the systematic gene disruption project | (Brachmann et al 1998) |
| ***Candida tropicalis*** | | |
| MY070362 | Clinical susceptible strain from INSPQ (Institut National de Santé Publique, Québec, Canada) | - |
| ***Candida krusei* (*Issatchenkia orientalis*)** | | |
| ATCC6258 | ATCC reference strain. Isolated from the sputum of patient with bronchomycosis | (Rudek 1978) |
| ***Candida parapsilosis*** | | |
| ATCC90018 | Reference susceptible strain used for CLSI antifungal susceptibility testing. Isolated from blood | (Espinel-Ingroff et al 1992) |
| ***Candida glabrata*** | | |
| ATCC90030 | Reference susceptible strain used for CLSI antifungal susceptibility testing. Isolated from blood | (Espinel-Ingroff et al 1992) |

**Table S1**. Fungal strains used in this study.

**Reference**

Brachmann CB, Davies A, Cost GJ, Caputo E, Li J, Hieter P *et al* (1998). Designer deletion strains derived from Saccharomyces cerevisiae S288C: a useful set of strains and plasmids for PCR-mediated gene disruption and other applications. *Yeast* **14:** 115-132.

Cruz MC, Goldstein AL, Blankenship J, Del Poeta M, Perfect JR, McCusker JH *et al* (2001). Rapamycin and less immunosuppressive analogs are toxic to Candida albicans and Cryptococcus neoformans via FKBP12-dependent inhibition of TOR. *Antimicrob Agents Chemother* **45:** 3162-3170.

Dunkel N, Blass J, Rogers PD, Morschhauser J (2008a). Mutations in the multi-drug resistance regulator MRR1, followed by loss of heterozygosity, are the main cause of MDR1 overexpression in fluconazole-resistant Candida albicans strains. *Mol Microbiol* **69:** 827-840.

Dunkel N, Liu TT, Barker KS, Homayouni R, Morschhauser J, Rogers PD (2008b). A gain-of-function mutation in the transcription factor Upc2p causes upregulation of ergosterol biosynthesis genes and increased fluconazole resistance in a clinical Candida albicans isolate. *Eukaryotic cell* **7:** 1180-1190.

Espinel-Ingroff A, Kish CW, Jr., Kerkering TM, Fromtling RA, Bartizal K, Galgiani JN *et al* (1992). Collaborative comparison of broth macrodilution and microdilution antifungal susceptibility tests. *J Clin Microbiol* **30:** 3138-3145.

Fonzi WA, Irwin MY (1993). Isogenic strain construction and gene mapping in Candida albicans. *Genetics* **134:** 717-728.

Franz R, Kelly SL, Lamb DC, Kelly DE, Ruhnke M, Morschhauser J (1998). Multiple molecular mechanisms contribute to a stepwise development of fluconazole resistance in clinical Candida albicans strains. *Antimicrob Agents Chemother* **42:** 3065-3072.

Franz R, Ruhnke M, Morschhauser J (1999). Molecular aspects of fluconazole resistance development in Candida albicans. *Mycoses* **42:** 453-458.

Jimenez-Ortigosa C, Paderu P, Motyl MR, Perlin DS (2014). Enfumafungin derivative MK-3118 shows increased in vitro potency against clinical echinocandin-resistant Candida Species and Aspergillus species isolates. *Antimicrob Agents Chemother* **58:** 1248-1251.

Morschhauser J, Barker KS, Liu TT, Bla BWJ, Homayouni R, Rogers PD (2007). The transcription factor Mrr1p controls expression of the MDR1 efflux pump and mediates multidrug resistance in Candida albicans. *PLoS Pathog* **3:** e164.

Rudek W (1978). Esterase activity in Candida species. *J Clin Microbiol* **8:** 756-759.

Saidane S, Weber S, De Deken X, St-Germain G, Raymond M (2006). PDR16-mediated azole resistance in Candida albicans. *Mol Microbiol* **60:** 1546-1562.
