# Supplementary material for: The Human Gut Microbial Metabolome Modulates Fungal Growth via the TOR Signaling Pathway
Source: mSphere. 2017 Dec 13;2(6):e00555-17. doi: 10.1128/mSphere.00555-17 (PMC5729221; doi:10.1128/mSphere.00555-17)
Supplement: TABLE S2 [file sph006172431st2.docx]

**Table S2.** List of GM-upregulated transcripts.

| **Gene** | **Fold induction** | **Description** | **Gene ID** |
| --- | --- | --- | --- |
| **Nitrogen metabolism** | | | |
| ***Permeases*** | | | |
| *OPT7* | 4.35 | Oligopeptide transporter | C4_00440C_B |
| *TPO3* | 16.49 | Polyamine transporter | C1_08790W_A |
| *MEP1* | 2.70 | Ammonium permease | C3_02310W_A |
| *MUP1* | 3.41 | Putative high affinity methionine permease | C1_11870W_A |
| *ALP1* | 14.12 | Cystine transporter | C1_10800C_A |
| *YPQ2* | 2.05 | Membrane transporter for cationic amino acids | C3_03690W_A |
| *SUL1* | 10.41 | Sulfate transporter | C4_02610C_B |
| ***Amino acid biosynthesis*** | | | |
| *ARG3* | 7.89 | Ornithine carbamoyltransferase | C6_03230W_A |
| *ARG5,6* | 2.37 | Arginine biosynthetic enzyme, processed into 2 polypeptides with acetylglutamate kinase (Arg6) activity and acetylglutamate-phosphate reductase (Arg5) activity | C1_09290C_A |
| *ARG8* | 3.36 | Putative acetylornithine aminotransferase | C4_05070C_B |
| *CPA2* | 2.28 | Putative arginine-specific carbamoylphosphate synthetase | CR_01330W_B |
| *IDP1* | 2.02 | Isocitrate dehydrogenase | C2_05890C_B |
| *MET2* | 4.22 | Homoserine acetyltransferase | CR_02170W_A |
| *MET10* | 3.85 | Sulfite reductase | C2_09140C_B |
| *MET15* | 4.46 | O-acetylhomoserine O-acetylserine sulfhydrylase | C4_00200C_B |
| *MET16* | 2.68 | Putative 3'-phosphoadenylsulfate reductase | C4_07030W_A |
| *HAL21* | 2.04 | Putative phosphoadenosine-5'-phosphate or 3'-phosphoadenosine 5'-phosphosulfate phosphatase | C6_00970C_B |
| *CYS1* | 11.74 | Protein similar to A. nidulans CysA serine O-trans-acetylase | C1_11610C_A |
| *HOM3* | 2.57 | Putative L-aspartate 4-P-transferase | C4_05540W_B |
| *DFR1* | 2.02 | Dihydrofolate reductase | C7_03130C_B |
| *HBR2* | 2.09 | Alanine glyoxylate aminotransferase | C6_04220C_B |
| *GDH3* | 6.38 | NADP-glutamate dehydrogenase | C4_06120W_B |
| *LYS12* | 2.47 | Homoisocitrate dehydrogenase | CR_01400W_A |
| *LYS22* | 5.33 | Homocitrate synthase | C2_04460W_B |
| *LYS4* | 2.80 | Homoaconitase | C4_04410C_B |
| *ILV5* | 2.21 | Ketol-acid reductoisomerase | C6_00870C_A |
| *C1_02970W_B* | 2.95 | Predicted aminotransferase based on *S. pombe* ortholog SPBC660.12c | C1_02970W_B |
| ***Amino acid utilization*** | | | |
| *NIT3* | 7.96 | Putative nitrilase | C1_10700C_A |
| *ALT1* | 2.74 | Alanine transaminase | C3_03480C_A |
| *BNA5* | 2.53 | Kynureninase | C1_08490W_B |
| *ARO10* | 5.53 | Aromatic decarboxylase | CR_06860C_A |
| *LAP3* | 3.39 | Putative aminopeptidase | CR_04480C_A |
| *CAR2* | 2.07 | Ornithine aminotransferase | C4_00160C_B |
| *CPA1* | 2.33 | Putative carbamoyl-phosphate synthase subunit | C4_01550C_A |
| *ADH2* | 2.75 | Alcohol dehydrogenase | C1_08330C_A |
| *FGR10* | 2.17 | Putative asparaginase | C4_04880W_B |
| ***Transcriptional regulators*** | | | |
| *GAT1* | 2.60 | GATA-type transcription factor; regulator of nitrogen utilization and NCR | C4_05880W_A |
| *NPR1* | 2.50 | Serine/threonine protein kinase, involved in regulation of ammonium transport | C1_06780W_B |
| **Carbohydrate metabolism** | | | |
| *PGM2* | 2.82 | Phosphoglucomutase | CR_02820W_A |
| *GLG21* | 5.05 | Glycogen synthesis initiator | C1_01360C_A |
| *UBC8* | 2.61 | Ubiquitin-conjugating enzyme that negatively regulates gluconeogenesis | C1_01830C_B |
| *MLS1* | 4.02 | Malate synthase | C1_09690W_A |
| *HXK2* | 2.44 | Hexokinase II | CR_04510W_A |
| *PYC2* | 2.89 | Putative pyruvate carboxylase | C4_03940C_B |
| *XKS1* | 4.12 | Xylulokinase | C4_05300W_A |
| *GLK1* | 4.77 | Glucokinase | CR_07150W_A |
| *GLK4* | 6.04 | Glucokinase | CR_07490C_A |
| *C4_02620C_A* | 2.20 | Carbohydrate kinase domain-containing protein | C4_02620C_A |
| *MDH1-1* | 2.69 | Malate dehydrogenase precursor | C4_01900C_A |
| *PHR1* | 2.56 | Cell surface glycosidase | C4_04530C_A |
| *CIT1* | 2.62 | Citrate synthase | CR_03500W_A |
| *DAK2* | 2.57 | Dihydroxyacetone kinase | C1_09190C_A |
| *GRE3* | 2.41 | D-xylose reductase | C5_02930C_A |
| *ADH2* | 2.75 | Alcohol dehydrogenase | C1_08330C_A |
| *ARA1* | 2.74 | D-Arabinose dehydrogenase | C2_08130W_A |
| *TPS2* | 2.97 | Trehalose-6-phosphate (Tre6P) phosphatase | C1_03380W_A |
| *HGT1* | 2.09 | High-affinity MFS glucose transporter | C1_01980W_B |
| *HGT18* | 2.09 | MFS glucose transporter | CR_03040C_A |
| *NAG3* | 54.83 | MFS transporter | C6_04610C_A |
| *NAG4* | 11.29 | MFS transporter | C6_04620C_B |
| *HGT20* | 8.11 | Putative glucose transporter of the major facilitator superfamily | C2_02610C_A |
| **Lipid and diverse metabolisms** | | | |
| *ICL1* | 20.63 | Isocitrate lyase; glyoxylate cycle enzyme | C1_04500W_B |
| *CRG1* | 9.70 | Methyltransferase involved in sphingolipid homeostasis | C6_02210W_B |
| *AMS1* | 7.28 | Putative alpha-mannosidase | C4_02360W_A |
| *DAP1* | 5.48 | Heme-binding protein; involved in regulation of cytochrome P450 protein Erg11p | CR_04060C_A |
| *FAA21* | 3.64 | Predicted acyl CoA synthetase | C3_02810C_A |
| *POF1* | 4.02 | Putative ATPase; predicted role in ER-associated protein catabolism | C1_13270W_B |
| *ATC1* | 3.87 | Cell wall acid trehalase | C1_06940C_B |
| *PHZ1* | 3.62 | Stationary phase enriched protein | C6_03320W_A |
| *INO4* | 3.49 | Transcription factor that regulates genes involved in phosphatidylcholine and phosphatidylinositol biosynthesis, fatty acid β-oxidation, and peroxisome biogenesis | C2_03840C_B |
| *YDC1* | 3.48 | Alkaline dihydroceramidase; involved in sphingolipid metabolism | C4_07050W_A |
| *PEX4* | 3.16 | Putative peroxisomal ubiquitin conjugating enzyme | C5_05430W_A |
| *MAK3* | 2.43 | Predicted peptide alpha-N-acetyltransferase | C4_01760W_A |
| *SNZ1* | 2.95 | Stationary phase protein; vitamin B synthesis | C1_02590C_B |
| *MUQ1* | 3.11 | Putative choline phosphate cytidylyltransferase/phosphoethanolamine cytidylyltransferase | C2_00260C_A |
| *PDX3* | 2.68 | Pyridoxamine-phosphate oxidase | CR_04590C_B |
| *DLD1* | 2.79 | Putative D-lactate dehydrogenase | C2_02980C_B |
| *RIB3* | 2.25 | 3,4-Dihydroxy-2-butanone 4-phosphate synthase | C1_12360C_B |
| *DES1* | 2.01 | Putative delta-4 sphingolipid desaturase | C5_05480W_B |
| *ERG7* | 2.20 | 2,3-epoxysqualene-lanosterol cyclase | C2_02460W_B |
| *LIP5* | 2.02 | Cold-activated secreted lipase | C7_02830C_B |
| *POX1-3* | 2.08 | Predicted acyl-CoA oxidase | C3_01960C_A |
| *HTD2* | 2.01 | Mitochondrial 3-hydroxyacyl-thioester dehydratase | C4_03200C_A |
| *TGL3* | 2.23 | Putative phospholipase of patatin family | C4_00950C_B |
| *ACH1* | 2.15 | Acetyl-coA hydrolase; acetate utilization | C5_02000C_A |
