# Supplementary material for: The Human Gut Microbial Metabolome Modulates Fungal Growth via the TOR Signaling Pathway
Source: mSphere. 2017 Dec 13;2(6):e00555-17. doi: 10.1128/mSphere.00555-17 (PMC5729221; doi:10.1128/mSphere.00555-17)
Supplement: TABLE S3 [file sph006172431st3.docx]

**Table S3.** List of GM-downregulated transcripts.

| **Gene** | **Fold induction** | **Description** | **Gene ID** |
| --- | --- | --- | --- |
| **Translation** | | | |
| **Ribosomal large subunit biogenesis** | | | |
| *RSA3* | -33.33 | Protein required for maturation of pre-60S ribosomal particles | C1_04710C_B |
| *REX4* | -3.13 | Putative RNA exonuclease | C1_12440W_A |
| *URB1* | -3.70 | Protein required for the normal accumulation of 25S and 5.8S rRNAs | C1_07660W_A |
| *RRP5* | -2.70 | RNA binding protein involved in synthesis of 18S and 5.8S rRNAs | C2_02540W_B |
| *NOP12* | -2.13 | Protein with role in maturation of LSU-rRNA from tricistronic rRNA transcript | C2_04120C_A |
| *EBP2* | -20.00 | Protein with mRNA binding activity, role in nuclear division, rRNA processing | C2_04570W_A |
| *PUF6* | -1.39 | Protein with mRNA 3'-UTR binding, translation repressor activity, nucleic acid binding activity | C2_05160C_B |
| *NOP53* | -1.52 | Protein with rRNA binding activity, role in maturation of LSU-rRNA from tricistronic rRNA transcript | C2_05750W_B |
| *RPR14* | -2.70 | Protein with role in ribosomal large subunit biogenesis, ribosomal small subunit biogenesis | C2_08180C_A |
| *NOP16* | -2.63 | Putative 66S pre-ribosomal particle component | C2_09660W_A |
| *PRP43* | -2.27 | Ortholog of S. cerevisiae Prp43, an RNA helicase in the DEAH-box family that functions in both RNA polymerase I and polymerase II transcript metabolism | C3_01560W_B |
| *RSA4* | -2.44 | Protein with a predicted role in ribosome biogenesis | C4_05010W_B |
| *RIX1* | -2.50 | Component of the Rix1 complex and possibly pre-replicative complexes; required for processing of ITS2 sequences from 35S pre-rRNA | C4_05230C_A |
| *MAK11* | -5.00 | Protein with a predicted role in 60S ribosomal subunit biogenesis | C4_05330C_B |
| *NUG1* | -3.45 | GTPase that associates with nuclear 60S pre-ribosomes; required for export of 60S ribosomal subunits from the nucleus | C4_06210C_B |
| *SQT1* | -11.11 | Protein with unfolded protein binding activity, role in ribosomal large subunit assembly | C5_05340W_A |
| *NOP2* | -1.85 | Protein with rRNA (cytosine-C5-)-methyltransferase activity and role in assembly of large subunit precursor of preribosome | CR_04170W_A |
| *GRC3* | -2.63 | Polynucleotide kinase present on rDNA | CR_06450W_A |
| *TIF6* | -2.17 | Protein with *S. cerevisae*/*S. pombe* Tif6; constituent of 66S pre-ribosomal particles | CR_07080W_B |
| *CSI2* | -2.50 | Protein with 66S pre-ribosomal particle component | C1_12310C_A |
| *DBP3* | -2.94 | Protein with ATP-dependent DEAD-box RNA helicase | C1_10030W_B |
| *ERB1* | -2.70 | Protein with role in ribosomal large subunit biogenesis | C1_04130W_A |
| *HAS1* | -3.70 | Functional homolog of S. cerevisiae Has1p, which is a nucleolar protein of the DEAD-box ATP-dependent RNA helicase family that is involved in biogenesis of the ribosome | C5_04750C_A |
| *HIT1* | -1.47 | Protein involved in C/D snoRNP assembly | C4_02750W_A |
| *JIP5* | -6.25 | Ortholog of S. cerevisiae Jip5; predicted role in biogenesis of the large ribosomal subunit | C1_08870C_A |
| *MRT4* | -33.33 | mRNA turnover protein | C6_02770W_A |
| *NIP7* | -1.96 | Nucleolar protein with role in ribosomal assembly | C6_02360W_B |
| *NOC2* | -1.35 | Nucleolar complex protein | CR_05520W_A |
| *NOP4* | -3.13 | Nucleolar protein | C1_04390C_A |
| *NOP8* | -5.26 | Ortholog of S. cereviiae Nop8; has a role in ribosomal large subunit biogenesis | C6_04330W_A |
| *NSA1* | -2.33 | 66S pre-ribosomal particles component | C2_07960C_A |
| *PES1* | -1.85 | Protein required for maturation of the large ribosomal subunit | C2_09320C_A |
| *REI1* | -2.33 | Cytoplasmic pre-60S factor | C1_05060W_A |
| *RPF1* | -4.00 | Nucleolar protein with a predicted role in the assembly and export of the large ribosomal subunit | C4_03270W_B |
| *RPF2* | -1.20 | pre-rRNA processing protein | C2_05230C_A |
| *RPL7* | -2.00 | Ribosomal protein L7 | CR_06120W_B |
| *RRP8* | -1.96 | Ribosomal protein |  |
| *RRS1* | -4.55 | Ribosome biogenesis and nuclear export protein | C1_00900W_A |
| *SDA1* | -2.38 | Nuclear protein involved in actin cytoskeleton organization, passage through Start, 60S ribosome biogenesis | CR_05660W_A |
| *SPB1* | -4.35 | AdoMet-dependent methyltransferase | C6_04160C_A |
| *SPB4* | -11.11 | ATP-dependent RNA helicase | C5_01600C_A |
| *YTM1* | -1.41 | Protein similar to S. cerevisiae Ytm1p, which is involved in biogenesis of the large ribosomal subunit | C1_09510W_A |
| *YVH1* | -1.96 | Dual specificity phosphatase | CR_03570C_A |
| **Ribosomal small subunit biogenesis** | | | |
| *BMS1* | -3.85 | Putative GTPase | C3_01000W_A |
| *NOP9* | -6.67 | U3-containing 90S preribosome subunit | C1_04040C_A |
| *ECM16* | -4.76 | Ortholog of S. cerevisiae Ecm16, an essential DEAH-box ATP-dependent RNA helicase specific to the U3 snoRNP required for 18S rRNA synthesis | C2_00410C_B |
| *SLX9* | -4.00 | Ortholog of Slx9 required for pre-rRNA processing; associated with 90S pre-ribosome and 43S small ribosomal subunit precursor, interacts with U3 snoRNA in S. cerevisiae | C2_06850W_A |
| *RRP14* | -2.70 | Protein with role in ribosomal large subunit biogenesis, ribosomal small subunit biogenesis and cytosolic large ribosomal subunit, nucleolus localization | C2_08180C_A |
| *KRI1* | -50.00 | Protein with role in endonucleolytic cleavage in ITS1 to separate SSU-rRNA from 5.8S rRNA and LSU-rRNA from tricistronic rRNA transcript | C3_02350W_A |
| *FAF1* | -3.33 | Protein with role in maturation of SSU-rRNA from tricistronic rRNA transcript (SSU-rRNA, 5.8S rRNA, LSU-rRNA) and cytoplasm, nucleolus localization | C4_05650W_A |
| C5_04910W_B | -1.43 | Protein required for maturation of 18S rRNA | C5_04910W_B |
| *DHR2* | -2.13 | DEAH-box ATP-dependent RNA helicase, required for 18S rRNA synthesis; rat catheter biofilm induced | C6_01040C_B |
| *TSR4* | -11.11 | Protein with role in maturation of SSU-rRNA and cytosol, nucleolus localization | C7_02930C_A |
| *CHR1* | -33.33 | DEAD-box ATP-dependent RNA helicase | C1_12600C_A |
| *TMA23* | -2.50 | Nucleolar protein | CR_01710W_A |
| *UTP23* | -3.23 | Protein with small ribosomal subunit rRNA binding activity | CR_02420W_A |
| *KRE33* | -1.75 | Ortholog of S. cerevisiae Kre33; essential; S. cerevisiae ortholog is essential and is required for biogenesis of the small ribosomal subunit | CR_04240C_A |
| *RRP36* | -2.00 | 90S preribosome component | CR_07030C_A |
| *DBP8* | -25.00 | Protein similar to S. cerevisiae Dbp8p, an ATP-dependent helicase involved in rRNA processing | CR_05630W_A |
| *DIP2* | -10.00 | Small ribonucleoprotein complex | C1_08290C_B |
| *ENP2* | -1.72 | Nucleolar protein | C7_03540C_A |
| *FYV5* | -1.37 | Protein with a predicted role maturation of 18S rRNA | C5_01480W_A |
| *IMP4* | -8.33 | SSU processome component | CR_07950W_A |
| *NOC4* | -3.23 | Nucleolar protein | C2_07340W_A |
| *NOP5* | -12.50 | Ortholog of S. cerevisiae Nop58; involved in pre-rRNA process | C6_00370C_A |
| *NOP6* | -3.13 | Putative ortholog of S. cerevisiae Nop6; role in ribosomal small subunit biogenesis | C1_06740C_A |
| *RCL1* | -50.00 | U3-containing 90S preribosome processome complex subunit | C2_07450C_A |
| *RPP1* | -3.45 | Putative ortholog of S. cerevisiae Rpp1; subunit of both RNase MRP and nuclear RNase P | C1_03800W_A |
| *RRS1* | -4.55 | Ribosome biogenesis and nuclear export protein | C1_00900W_A |
| *SAS10* | -6.25 | U3-containing small subunit processome complex subunit | C4_02790C_A |
| *TSR2* | -1.23 | Protein with a predicted role in pre-rRNA processing | C1_03060C_A |
| *UTP13* | -5.00 | U3 snoRNA-associated protein | C5_02550C_A |
| *UTP20* | -5.56 | snoRNA-binding protein | C3_01200W_A |
| *UTP4* | -2.78 | U3 snoRNA-associated protein | C3_02130W_A |
| **Ribosome localization** | | | |
| *ECM1* | -1.59 | Pre-ribosomal factor | C4_04100C_A |
| *NMD3* | -2.44 | Nonsense-mediated mRNA decay protein | CR_06720W_A |
| *BUD20* | -4.35 | C2H2-type zinc finger protein required for ribosome assembly | C1_02450C_A |
| *NOG2* | -2.94 | Nucleolar GTPase | C6_03640W_A |
| **snRNA, snoRNA and tRNA processing** | | | |
| *TRM3* | -2.13 | tRNA (guanine) methyltransferase activity | C4_03830W_A |
| *SEN2* | -2.44 | tRNA splicing endonuclease subunit | C4_02640C_A |
| *POP7* | -4.35 | Protein with RNA binding, ribonuclease MRP activity, ribonuclease P activity | C4_00740W_A |
| *POP4* | -1.92 | Ortholog of S. cerevisiae Pop4; a subunit of both RNase MRP and nuclear RNase P | C6_02910W_A |
| *TRM5* | -50.00 | Protein with tRNA (guanine-N1-)-methyltransferase activity, role in mitochondrial tRNA methylation, tRNA N1-guanine methylation and mitochondrial matrix | C4_03730C_B |
| *DEG1* | -33.33 | Protein with pseudouridine synthase activity, role in mRNA pseudouridine synthesis, tRNA pseudouridine synthesis and cytosol | C5_01610W_A |
| *TGS1* | -3.13 | Protein with RNA methyltransferase activity, role in 7-methylguanosine cap hypermethylation, meiotic nuclear division, nucleologenesis, regulation of telomere maintenance via telomerase, tRNA processing and nucleolus localization | C2_07360W_A |
| *DUS1* | -3.85 | tRNA dihydrouridine synthase | C2_09500W_A |
| *NOP1* | -3.70 | Nucleolar protein | C4_06720W_A |
| *SMM1* | -4.76 | Dihydrouridine synthase | C2_09890W_A |
| *RPP1* | -3.45 | Subunit of both RNase MRP and nuclear RNase P | C1_03800W_A |
| *PUS7* | -3.03 | Pseudouridine synthase; catalyzes pseudouridylation in U2 snRNA, 5S rRNA, cytoplasmic tRNAs and in pre-tRNA(Tyr) | C2_10270W_A |
| *RRP40* | -2.78 | Exosome non-catalytic core component; involved in 3'-5' RNA processing and degradation in the nucleus and cytoplasm | C1_01160C_A |
| *DEG1* | -33.33 | Protein with pseudouridine synthase activity, role in mRNA pseudouridine synthesis, tRNA pseudouridine synthesis | C5_01610W_A |
| *DUS3* | -3.33 | Dihydrouridine synthase | C2_02420C_A |
| *NHP2* | -3.23 | Putative H/ACA snoRNP protein | CR_04360C_A |
| *MTR4* | -1.92 | Ortholog of S. cerevisiae Mtr4, an ATP-dependent 3'-5' RNA helicase of the DEAD-box family; Hap43-induced gene | C7_03400C_A |
| *RRP6* | -20.00 | Putative nuclear exosome exonuclease component; | C1_05050C_A |
| *LRP1* | -6.67 | Nuclear exosome-associated nucleic acid binding protein | C1_07960W_A |
| *NAF1* | -4.55 | RNA-binding protein; role in assembly of box H/ACA snoRNPs and thus pre-rRNA processing | CR_04110W_A |
| *GAR1* | -11.11 | H/ACA snoRNP pseudouridylase complex protein; | C1_11550W_A |
| *RRP46* | -1.96 | Protein with role in exonucleolytic trimming to generate mature 3'-end of 5.8S rRNA from tricistronic rRNA transcript | C2_06660W_A |
| **Transcription from RNA polymerase I and III promoters** | | | |
| *RPA43* | -4.76 | Protein with RNA polymerase I activity, role in transcription of nuclear large rRNA transcript from RNA polymerase I promoter and DNA-directed RNA polymerase I complex | CR_01950W_B |
| *RPA12* | -4.35 | DNA-directed RNA polymerase I | C2_07300C_B |
| *RPA190* | -5.88 | RNA polymerase I subunit A190 | C1_10670C_B |
| *PZF1* | -3.70 | Transcription factor IIIA (TFIIIA); essential DNA binding protein required for transcription of 5S rRNA by RNA polymerase III | C2_04860W_A |
