# Supplementary figures and images for: The Human Gut Microbial Metabolome Modulates Fungal Growth via the TOR Signaling Pathway
Source: mSphere. 2017 Dec 13;2(6):e00555-17. doi: 10.1128/mSphere.00555-17 (PMC5729221; doi:10.1128/mSphere.00555-17)

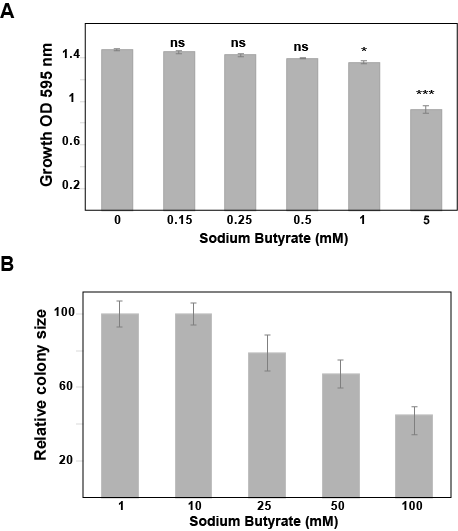

Supplement: FIG S1 [file sph006172431sf1.tif]
